# Role of Paralogue of XRCC4 and XLF in DNA Damage Repair and Cancer Development

**DOI:** 10.3389/fimmu.2022.852453

**Published:** 2022-03-02

**Authors:** Jialin Tang, Zhongxia Li, Qiong Wu, Muhammad Irfan, Weili Li, Xiangyu Liu

**Affiliations:** ^1^ Guangdong Key Laboratory of Genome Instability and Human Disease Prevention, Department of Biochemistry and Molecular Biology, Shenzhen University School of Medicine, Shenzhen, China; ^2^ Department of Hematology, The Second People’s Hospital of Shenzhen, Shenzhen, China

**Keywords:** DSBs, XRCC4, XLF, PAXX, cNHEJ, cancer, posttranslational modifications

## Abstract

Non-homologous end joining (cNHEJ) is a major pathway to repair double-strand breaks (DSBs) in DNA. Several core cNHEJ are involved in the progress of the repair such as KU70 and 80, DNA-dependent protein kinase catalytic subunit (DNA-PKcs), Artemis, X-ray repair cross-complementing protein 4 (XRCC4), DNA ligase IV, and XRCC4-like factor (XLF). Recent studies have added a number of new proteins during cNHEJ. One of the newly identified proteins is Paralogue of XRCC4 and XLF (PAXX), which acts as a scaffold that is required to stabilize the KU70/80 heterodimer at DSBs sites and promotes the assembly and/or stability of the cNHEJ machinery. PAXX plays an essential role in lymphocyte development in XLF-deficient background, while XLF/PAXX double-deficient mouse embryo died before birth. Emerging evidence also shows a connection between the expression levels of PAXX and cancer development in human patients, indicating a prognosis role of the protein. This review will summarize and discuss the function of PAXX in DSBs repair and its potential role in cancer development.

## Introduction

DNA double-strand breaks (DSBs) create harmful lesions that are typically generated in response to extrinsic sources like ionizing radiation (IR) and chemotherapeutic drugs or intrinsic sources such as DNA replication fork collapse, transcription, and oxidative stress. If the DSBs are not efficiently joined, unrepaired DNA ends could lead to severe consequences such as cell cycle arrest, apoptosis, or senescence. On the other hand, if the DSB ends are joined improperly, mis-repaired DNA ends could lead to abnormalities such as genomic instability, chromosome translocation, and subsequent carcinogenesis (1–5). In eukaryotic cells, two major pathways are responsible for the repair of DSBs: homologous recombination (HR) and classical non-homologous end-joining (cNHEJ). HR uses an intact DNA as a template to accurately repair the breaks to keep the fidelity of the genome. HR is considered to be mediated by a couple of factors including the single-strand binding protein RPA and the human homologs of *Saccharomyces cerevisiae* Rad51 (6). Due to the requirement of the sister chromatid or a homologous chromosome that provides the template, HR is generally restricted to late S or G2 cell cycle (7). cNHEJ is the other major DNA repair pathway in eukaryotic organisms including humans (4, 8–14). Characteristically, cNHEJ directly joins broken ends together and does not require a homologous template; thus, it is not restricted to a certain phase of the cell cycle (15). Recently, the alternative end-joining (Alt-EJ) is emerging as another important repair pathway that describes end joining events lacking cNHEJ factors.

The general mechanism of cNHEJ is the recognition of DSBs and bridging of the broken ends, assembly, and stabilization of different repair factors at the damaging sites, and the dissolve of these factors after the repair (4, 15). cNHEJ comprises core KU70 and KU80 subunits that form the KU heterodimer, which recognizes double-strand DNA ends and promotes the recruitment of downstream cNHEJ core factors, including the nuclear serine/threonine kinase DNA-dependent protein kinase catalytic subunit (DNA-PKcs), Artemis nuclease, X-ray repair cross-complementing protein 4 (XRCC4), XRCC4-like factor (XLF), and DNA ligase IV (LIG4). In recent years, several new factors were discovered to play crucial roles in cNHEJ such as modulator of retroviral infection (Mri) and Paralogue of XRCC4 and XLF (PAXX) (16).

Interestingly, DNA repair factor 53BP1, DNA damage response kinases ATM and DNA-PKcs, the histone variant H2AX, Snf2-family helicase-like ERCC6L2, the RNF8 and RNF168 ubiquitin ligases, the mediator of DNA damage checkpoint protein 1 (MDC1), and RAG2 were found to have redundant roles with cNHEJ factor XLF, even though single deficiency of either protein did not abolish cNHEJ (17–22). Once the DSBs are generated, the KU heterodimer and DNA-PKcs form the DNA-PK holoenzyme that associates with the DNA ends. DNA-PK autophosphorylates DNA-PKcs to license further ligation activities (23, 24). Artemis nuclease is essential for opening loops, bubbles, flaps, gaps, and hairpins, which are obstacles for ligation (25–27). Activation of Artemis requires both the DNA-PKcs protein and the kinase activity of either DNA-PKcs or ATM (24). XRCC4 and PAXX are requisite for the stabilization and activation of LIG4, which essentially ligates the DNA ends (28–30). XLF stimulates the ligase activity by interacting with XRCC4-LIG4 (31), while Mri prevents components of HR or A-EJ pathway from access of broken DNA ends (32).

Out of these core NHEJ factors, PAXX is relatively a new member of XRCC4 and XLF-superfamily that possess an identical structure of N-terminal domain. It is widely distributed in all human body with low tissue specificity, despite that it is highly expressed in the blood dendritic cells and lymphocytes such as B and T cells, according to the database of The Human Protein Atlas. In addition, *Paxx* gene exists in all vertebrates but not in most invertebrates or yeast. This evolutionary distribution of *Paxx* is different from *Xrcc4* or *Xlf* that is conserved from yeast to vertebrates (33). However, *Paxx* seems to have co-evolved with *Prkdc* gene that encodes DNA-PKcs protein and *Dclre1c* gene that encodes Artemis protein, which are restricted to higher eukaryotes (34), suggesting that PAXX may function in the same manner as DNA-PKcs and Artemis, connecting complex rather than simple DNA ends (35). On the basis of experimental data, it has been proven that even though PAXX itself does not bind DNA, it directly interacts with the KU complex and promotes KU-dependent DNA ligation (33, 36–38). However, the underlying mechanism how PAXX associates with cNHEJ still needs to be further explored.

**Figure 1:**
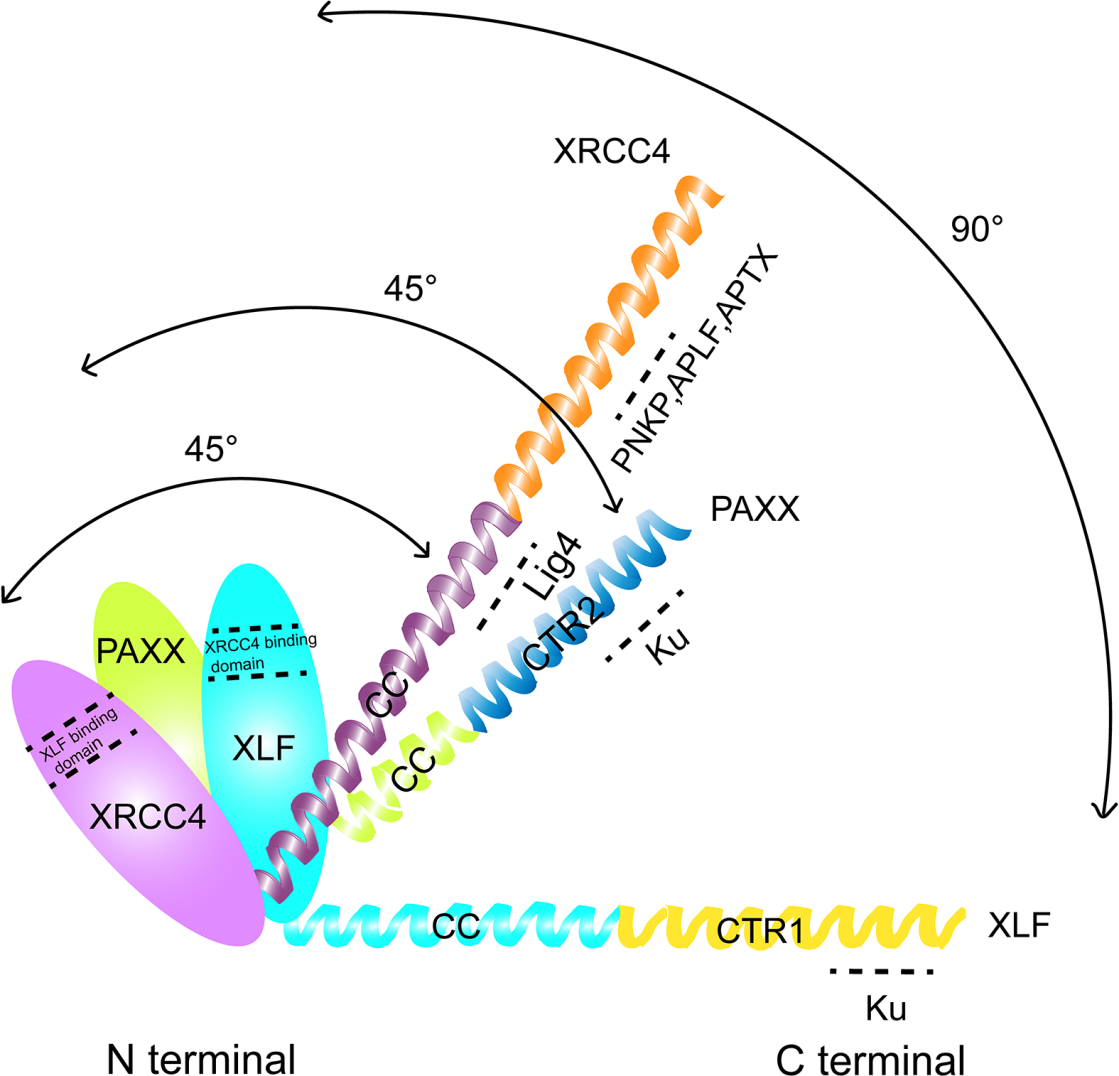
Comparison of the structural domains of PAXX, XLF, and XRCC4. The interacting domain of each protein with other factors were indicated.

The human PAXX protein contains a total of 204 amino acids. Seven beta-strands (S1–S7) and three helixes (H1–H3) are observed in the N-terminus of PAXX protein ranging from the 1st to 145th amino acid, in which a spherical head domain and a coiled-coil stalk make the protein primarily as a dimer in solution. This structure is similar to the dimeric interface formed by XRCC4, XLF, and SAS6 (33, 39–43) (**Figure 1**). Interestingly, despite that PAXX is called “Paralogue of XRCC4 and XLF,” it shows more structural similarities with XRCC4 than XLF. In the case of XLF, the C-terminus of the XLF is folded back to the junction between H3 and the head domain, forming a 90°C angle between them. However, the angle between the helix H3 and the head field of XRCC4 and PAXX is about 45°C due to lack of tension. Different from XRCC4, PAXX has a much shorter coiled-coil stalk. Since LIG4 binding requires an extended stem of XRCC4, it implies that PAXX protein may not be able to directly interact with LIG4 (33). Besides, the C-terminal domains (CTRs) of the XRCC4, XLF, and PAXX superfamily are more diverse with different functions. Deletion (AA1–170) or point mutation (RRR177–179AAA, IN186–187AA, or F201A) of the PAXX CTRs resulted in loss of its interaction with KU. An electrophoretic mobility shift assay (EMSA) showed that V199A/F201A mutants of PAXX abrogated its binding with Ku molecules *in vitro*. In addition, the F201A mutation compromised the nuclear localization of PAXX. These results suggested that the C-terminus of the PAXX might be the regulatory region of the protein, with the underlying mechanism to be further explored (33, 43).

Interestingly, hypomethylation of *Paxx* gene promoter and higher expression level of mRNA were observed in tumors compared with normal tissues, implying that overexpression of PAXX might be correlated with tumorigenesis. In summary, given the importance of this new cNHEJ factor, we will discuss the role of PAXX in DNA repair and its biological functions in lymphocyte development and tumorigenesis in this review.

## PAXX and DNA Repair

To explore the role of PAXX in DNA repair, PAXX-deficient chicken DT40 cells were treated with different sources of DNA-damaging agents, including irradiation (IR), bleomycin, etoposide (VP16), ICRF193, and camptothecin (CPT). Cell survival assay showed that PAXX deficiency increased sensitivity to most of these stimuli except for CPT (33), the inhibitor of DNA topoisomerase I. Human colon cancer cell line HCT116 in which PAXX was depleted using CRISPR-Cas9 technology or osteosarcoma cell line U2OS that was treated with small interfering RNA (siRNA) against PAXX also showed hypersensitivity to IR treatment and delayed γH2AX foci resolution (43). Mouse B-lymphocyte cell line CH12F3 in which PAXX was knocked out was hypersensitive to zeocin, but its class switch recombination (CSR) efficiency was not affected (44). Among these agents mentioned above, CPT induces replication-dependent DNA damage that relies on HR to repair (45). In contrast, repair of ICRF193 caused DNA damage replies more on cNHEJ rather than HR pathway. In addition, G1 arrested *Paxx* ^−/−^ cells were more sensitive to IR, compared with unsynchronized cells, suggesting that PAXX played a unique role in G1 phase. Moreover, PAXX-deficient U2OS or human retinal pigment epithelial RPE-1 cells were defective in random-plasmid integration, which was dependent on cNHEJ (43). All this evidence suggests that PAXX plays an essential role in cNHEJ, and we will further discuss the mechanism that PAXX plays roles in DNA repair in the next few paragraphs.

## Role of PAXX in cNHEJ Repair Pathway

### PAXX Associates With KU Complex

*In vitro* and *in vivo* experiments showed that PAXX bound in the core region of KU heterodimer. Even though PAXX also associated with other cNHEJ factors such as XRCC4, the interaction between PAXX and KU80 was more resistant to high salt treatment than those between PAXX and other cNHEJ factors. Immunoprecipitation (IP) experiments using endogenous antibodies confirmed that PAXX and KU80 interacted with each other (33). To find out the mechanism of the interaction, Ochi et al. synthesized a biotinylated peptide containing PAXX residues 177–204 and performed a peptide pulldown assay. The WT but not the peptide containing V199A/F201A was able to pull down KU heterodimer (43). As KU70 alone bound to DNA without KU80, Tadi et al. accessed the binding of PAXX with KU70-DNA complex *in vitro* and found that PAXX formed a stable ternary complex with KU70-DNA. EMSA assay showed that PAXX but not its mutant V199A or F201A supershifted the KU-DNA complex. Correspondingly, *in vitro* end ligation experiment showed that WT PAXX but not V199A/F201A mutant markedly stimulated DNA ligation in reactions containing both the XRCC4/LIG4 complex and KU complex (43). The presence of PAXX and KU greatly increased the blunt end ligation efficiency of LIG4/XRCC4 by hundreds of times but only slightly promoted the ligation of cohesive ends (~1.2 times). In addition, binding of PAXX to Ku promoted DSB repair at the biochemical and cellular levels (33, 43).

Interestingly, a combination of PAXX and KU-DNA complex requires a DNA length >30 bp, while the binding site of KU and DNA is only 14 bp (46), suggesting that the combination of PAXX and KU-DNA complex requires free DNA extension to stabilize the ternary complex. Ochi et al. also proved that PAXX regulated the end ligation efficiency by binding to the protruding ends of KU-bound DNA *in vitro*. The requirement of the DNA extension could be double-strand DNA, or either 3′ or 5′ single-strand DNA, or even a poly(dT) ssDNA tail. Immunofluorescence (IF) analysis in mouse embryonic fibroblasts (MEF) cells showed that knockout of KU80 significantly reduces the recruitment of PAXX at the DSBs. Both full-length KU80 and a truncated KU80 lacking DNA-PKcs binding domain fully restored PAXX recruitment, suggesting that PAXX and DNA-PKcs did not compete the C-terminus of KU80. Interestingly, deficiency of PAXX can also reduce the accumulation of KU at the DSBs (47). However, since PAXX does not directly bind DNA, it could not prevent KU’s translocation from DNA ends to more inward position on the DNA (38). All these data suggested that PAXX and KU have a very close connection, and several important functions of PAXX in DNA repair is dependent on KU protein.

### PAXX and XLF

Endogenous immunoprecipitation assay did not detect direct interaction between PAXX and XLF. Nevertheless, PAXX and XLF double-deficient cells showed hypersensitivity to VP16 and doxorubicin, compared with WT, *Paxx^−/−^* or *Xlf^−/−^* cells. Consistently, even though PAXX or XLF single-deficient mice develop normally, PAXX/XLF double deficiency caused mouse embryonic lethality before E19.0 (36, 47, 48). A higher caspase activity have been detected in both neural progenitor cells and post-mitotic neurons in PAXX/XLF double-deficient mice, suggesting that the level of apoptosis was significantly increased, which led to neurodevelopmental defects (47, 48). MEF cells from E14.5 *Xlf^−/−^Paxx^−/−^* embryos were generated to access the potential cause of the embryonic lethality. *Xlf^−/−^Paxx^−/−^* MEFs displayed ~25% reduction in the percentage of S phase and failed to thrive early passages. Consistently, Xlf/Paxx^−/−^ MEFs were sensitive to IR or VP16, but not to hydroxyurea, a replication stress inducer. In addition, Xlf/Paxx^−/−^ MEFs showed severe genome instability with almost all of the cytogenetic abnormalities being chromosomal breaks, similar to those observed in *Xrcc4* ^−/−^ cells (47). γH2AX was commonly used as a marker for DNA damage to monitor the repair of genotoxic DSBs (49). G1-arrested WT, *Paxx^−/−^*, *Xlf^−/−^*, *Xlf^−/−^Paxx^−/−^*, and *Lig4* ^−/−^ Eμ -Bcl2+ Abelson murine leukemia virus (A-MuLV)-transformed Abl pre-B cells from mice were treated with bleocin or VP16. After 24 h recovery, similar to *Lig4* ^−/−^ control, *Xlf* ^−/−^ *Paxx* ^−/−^ pre-B cells showed persistent γH2AX foci compared with WT or single-deficient cells, implying that PAXX/XLF double deficiency, instead of single deficiency of each protein, blocked repair of genotoxic DSBs (49).

**Figure 2:**
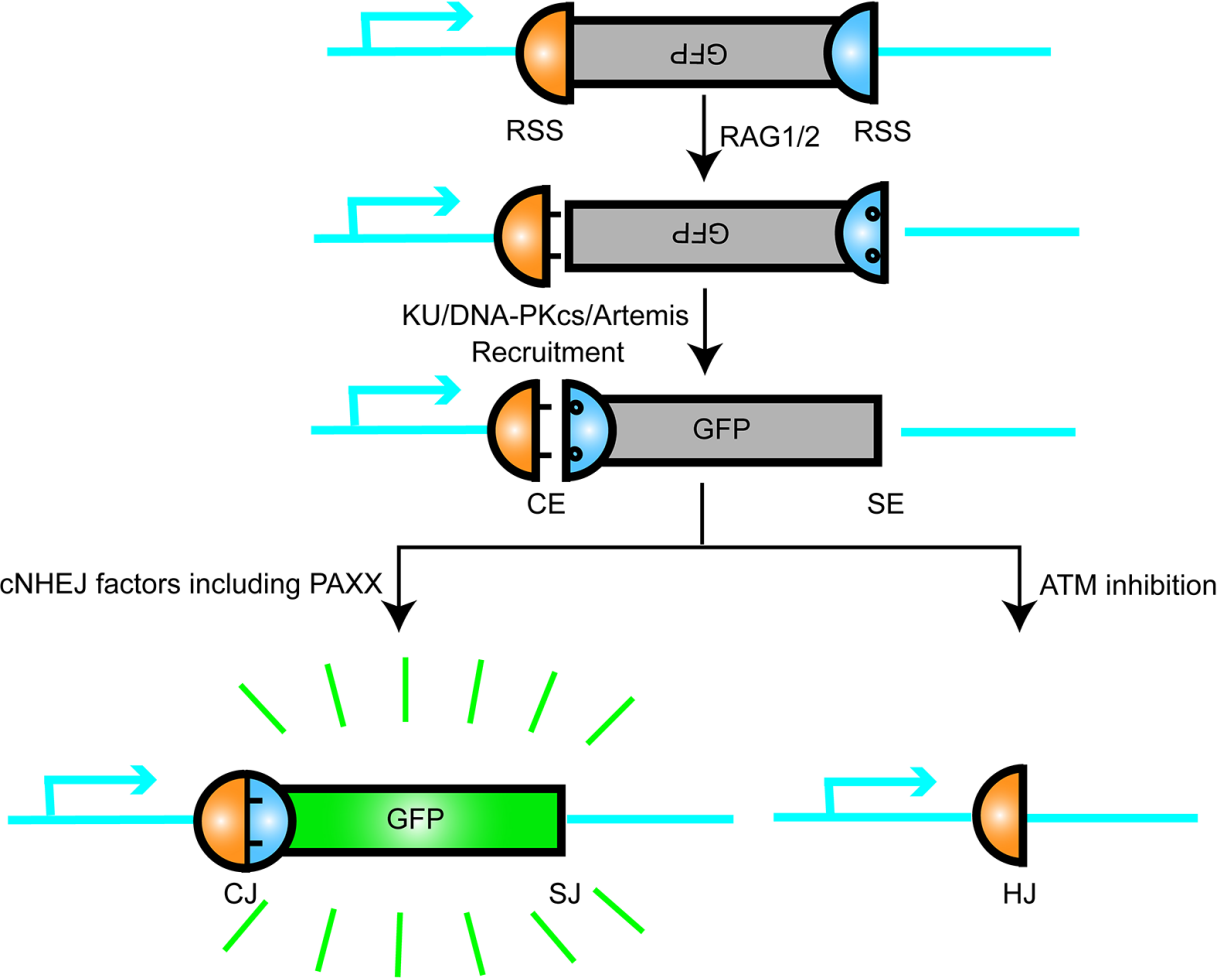
Schematic representation of V(D)J recombination. RAG1/2 nuclease introduce DSB at recombination signal sequences (RSS) and generates a pair of blunt signal ends (SEs) and a pair of covalently sealed hairpined coding ends (CEs). The hairpin CEs were opened by DNA-PKcs/Artemis and then joined by the cNHEJ factors including PAXX, so that the inverted GFP sequence were flipped and turned on. This model is like a plug was connected and a green bulb was lighted up.

The chromosomal V(D)J recombination assay in Abl pre-B cell lines was performed to further characterize the function of PAXX and XLF in cNHEJ. The assay was initiated by a v-abl kinase inhibitor STI571 (Imatinib) that induced G1 cell cycle arrest in the pre-B cells. In this case, RAG endonuclease was accumulated, and then, the recombination signal sequences (RSS) was cleaved at a chromosomal integrated pMX-INV inversional substrate. Since pMX-INV contained an inverted green fluorescent protein (GFP) cassette flanked by RSS, the GFP cassette could be inverted back to the same orientation and expressed the GFP fluorescent signal as the promoter by cNHEJ-mediated repair (50–52) (**Figure 2**). This assay clearly showed no GFP expression in *Xlf^−/−^Paxx^−/−^* cells after STI571 treatment, similar to those seen in *Xrcc4^−/−^* cells after STI571 induction, indicating end-ligation defects in *Xlf^−/−^Paxx^−/−^* cells. Southern blot assay also showed accumulation of signal ends (SEs) and coding ends (CEs) fragments. Notably, different from what was observed in *Xlf^−/−^53bp1^−/−^* cells, SEs and CEs did not appear to be resected in *Xlf^−/−^Paxx^−/−^* cells, suggesting that PAXX was less likely to be required to protect broken DNA ends. Moreover, DSBs generated by I-PpoI nuclease were also not able to be repaired in *Xlf^−/−^Paxx^−/−^* MEF cells. In conclusion, these results suggested that PAXX and XLF synergistically function in cNHEJ, at least in the mouse recombining lymphocytes and MEF cells (47, 53, 54).

Although PAXX and XLF participate redundantly in cNHEJ, and the two molecules share structural similarities, they have distinct functions (47). For example, XLF can stimulate the ligation of mismatched and non-cohesive ends *in vitro*, while PAXX cannot. PAXX alone could promote blunt end ligation in a KU-LIG4/XRCC4-dependent manner (38).Interestingly, in the presence of XLF, PAXX stimulated the ligation of non-cohesive ends but not the blunt ends (33, 55). *In vivo*, T cells of *Paxx* ^−/−^ mice developed normally, while XLF knockout mice have a slight reduction in lymphocyte numbers and an increase in thymocyte apoptosis (56, 57). In addition, while *Xlf^−/−^* cells require both ATM and DNA-PKcs kinase activity for efficient V(D)J recombination (51, 58). PAXX had no obvious functional redundancy with ATM or DNA-PKcs, and inhibition of ATM or DNA-PKcs activity in *Paxx* ^−/−^ Abl pro-B cells exerted limited effect on V(D)J recombination. *Paxx^−/−^Atm^−/−^* mice showed no difference with *Atm^−/−^* mice in phenotype (36). Similarly, PAXX did not seem to have redundant roles with MRI, while MRI and XLF deficiency exhibit embryonic lethality (22, 32). Moreover, overexpression of PAXX was not able to rescue the end-ligation defects in XLF-deficient cells with ATM inhibition (47). All these results indicate that PAXX has distinct functions with XLF in cNHEJ, and PAXX and XLF may synergistically regulate cNHEJ in different mechanisms.

Interestingly, synthetic lethality between XLF and PAXX was rescued by inactivation of tumor suppressor p53 (59). Since XRCC4/p53 double knockout mice developed pro-B-cell lymphomas with IgH-c-myc chromosomal translocations, it is interesting to see whether XLF/PAXX/p53-deficient mice also develop lymphoma that is reminiscent to XRCC4/p53 double knockout mice (60).

### PAXX and DNA-PKcs

One of the core events of cNHEJ is the recruitment of DNA-PKcs by KU heterodimer to the broken DNA ends to form the DNA-PK holoenzyme, which is then activated by autophosphorylation and promotes the DSBs repair mechanism onset (61, 62). DNA-PKcs is functionally redundant with ATM, and ATM is functionally redundant with XLF (51, 63, 64). Therefore, it is reasonable to explore the functional correlation between DNA-PKcs and XLF or XRCC4 homologs such as PAXX. In fact, DNA-PKcs and XLF are functionally redundant in mice. While DNA-PKcs-deficient mice were born in Mendelian ratio despite with severe combined immunodeficiency (SCID), the ratio of births of the XLF/DNA-PKcs double knockout mice did not conform to the Mendelian ratio, and the survival time of the mice did not exceed 10 days (58, 65–68). In addition, increased levels of abnormal metaphases were observed in *Xlf^−/−^DNA-PKcs^−/−^* fibroblasts, which were almost twice of that in *Xlf^−/−^* cells or *DNA-PKcs* ^−/−^ cells (58). However, in the case of PAXX and DNA-PKcs, the sensitivity to etoposide in PAXX/DNA-PKcs double knockout human HAP1 cells is the same as that in DNA-PKcs knockout cells alone (69). The telomere fluorescence *in situ* hybridization (T-FISH) assay to measure the chromatin fragmentation of these single- or double-knocked cells also showed that the level of genome instability of PAXX/DNA-PKcs double-knocked cells was close to that of DNA-PKcs knockout cells alone (69). In addition, DNA-PKcs kinase inhibition by NU7741 inhibitor in PAXX-deficient Abelson cells did not block the formation of CJ products or the expression of GFP signals (47). These experimental results suggest that while both PAXX and DNA-PKcs are recruited to the DNA ends by KU and each play critical roles, they do not seem to have redundant functions in cNHEJ. Consistently, double knockout mice deficient for PAXX and DNA-PKcs were born without detectable defects and were indistinguishable from the *Dna-pkcs* ^−/−^ mice (59).

### PAXX and XRCC4/LIG4 Complex

LIG4 is the most important DNA ligase during the progress of cNHEJ (70). The stability and activity of LIG4 depend on XRCC4 that forms a ligation complex with LIG4 to significantly improve the ligation efficiency. Therefore, the DNA end ligation of cNHEJ is largely dependent on the stability and activity of the XRCC4-LIG4 complex (28, 71). As an XRCC4-like factor, XLF directly interacts with XRCC4-LIG4 complex to improve the efficiency of DSBs ligation (72, 73). Does PAXX play a similar role in the XRCC4-LIG4 complex activity?

A direct evidence from live cell imaging experiment showed that the deficiency of PAXX in MEF cells moderately affected GFP-LIG4 recruitment (47). Interestingly, as previously discussed, ligation assay using blunt-ended DNA showed that PAXX itself cannot stimulate the activity of XRCC4-LIG4 complex, but when KU was present, the ligation activity of blunt-ended DNA was significantly increased, indicating that PAXX is dependent on KU to stimulate the XRCC4-LIG4 activity (38, 55). However, this is different from the previous report that PAXX had no significant effect on the ligation of *Eco*RV generated blunt-ended DNA, no matter whether XLF was added or not in the system (33). This difference is probably caused by the different concentrations of protein used in each reaction. In addition, ligation assay using cohesive DNA ends showed that PAXX itself had almost no stimulating effect on XRCC4-LIG4 complex (38). In this case, XLF is the dominant factor that is essential for the sufficient ligation of cohesive DNA ends. Instead, PAXX stimulates the ligation of non-cohesive DNA ends in the presence of XLF (33).

These results suggest that PAXX is more likely an accessory factor that associates with KU, and its function in cNHEJ is redundant with XLF (38). Therefore, effective protein interaction omics analysis could be used to identify the differences in the PAXX-associated interaction networks to further explore the bona fide function of PAXX in the future. Furthermore, although the stimulation of XRCC4-LIG4 complex by PAXX seems to be less important than XLF *in vitro*, whether PAXX has a stimulating effect for XRCC4-LIG4 complex under special processes under physiological conditions remains unclear.

### PAXX and Polymerases λ

The fact that PAXX possesses an XLF-dependent stimulatory effect on the ligation of non-cohesive DNA ends (33) implies that PAXX may play a special role in dealing with non-compatible DNA ends. Mass spectrometry and IP assay confirmed that Pol λ, which is a novel DNA polymerase that belongs to family X, was as an abundant PAXX-interacting protein. Importantly, IP of endogenous Pol λ showed that PAXX, XLF, and XRCC4 co-purified with Pol λ. The interaction of PAXX with Pol λ is DNA independent but requires DNA-bound KU. In addition, the N-terminal BRCT domain of Pol λ is necessary for its interaction with PAXX (37).

Previously, Pol λ has been shown to localize to oxidative DNA lesions and protected cells against oxidative stress (74), and *in vitro* experiment showed that Pol λ catalyzed gap-filling synthesis. Since Pol λ was co-purified with PAXX, immunofluorescence assay was performed to access the recruitment of Polλ at micro-irradiation-induced damaging sites. EGFP or mCherry tag fused N-termini of Pol λ was recruited to laser-induced DSBs following laser-induced DNA damage. Interestingly, the recruitment of EGFP-Pol λ fusion protein was substantially reduced in PAXX-deficient U2OS cells, suggesting that PAXX plays a critical role in recruiting Pol λ to DSBs (37). Besides, PAXX and other XRCC4 family proteins stimulated Pol λ gap-filling activity. Fragmentation of PAXX showed that head domain of PAXX (1–113 aa) was required for Polλ-dependent gap-filling activity (37). Surprisingly, mutant PAXX protein that was defective in interacting with DNA-bound KU enhanced Pol λ-dependent gap-filling synthesis comparable to WT PAXX, implying that KU was not required for PAXX to stimulate Pol λ activity.

Furthermore, to understand how Pol λ functions with PAXX and other XRCC4 family proteins, Pol λ was knocked down using siRNA to monitor the IR sensitivity. As Pol λ knockout in WT cells resulted in weak IR sensitivity, defects of Pol λ in *Paxx^−/−^* cells generated hypersensitivity. Interestingly, in PAXX/XLF knockout cells, which were already hypersensitive to IR, depletion of Pol λ did not lead to significant difference in radiosensitivity. These observation indicates that Pol λ functions as a key functional mediator in a common pathway of PAXX and XLF (37).

## Role of PAXX in Other Repair Pathways

As discussed extensively above, PAXX contributes to cNHEJ by interacting with several cNHEJ-related factors and promotes DSB repair through cNHEJ pathway (33, 43). Mouse model also confirmed that PAXX played a critical role in cNHEJ that was masked by XLF (36, 47). In-depth study showed that PAXX and XLF play distinct roles in cNHEJ (36, 47, 49, 53, 54).

To find out whether PAXX played a role in other parallel DNA repair pathways independent of cNHEJ, such as HR or base excision repair (BER), Yang et al. used plasmid-based cNHEJ and HR reporter assays to detect the cNHEJ and HR efficiency. They observed that in PAXX-deficient U87 cells, HR efficiency was increased by 18%–21% (75). However, since deficiency of cNHEJ factors could significantly enhance HDR (76), whether PAXX plays a direct role in HR still needs to be explored.

Interestingly, in a temozolomide (TMZ)-resistant U87 cell line, PAXX protein level was increased, implying that PAXX might contribute to TMZ resistance in glioma cell line (75). TMZ can alkylate DNA and add a methyl group to purine base to introduce O6-methylguanine (O6-MeG) specifically (77). Notably, over 80% of DNA damage caused by TMZ are N-methylated bases that are substrates for BER pathway. This means that BER may be responsible for TMZ resistance, at least partially, but importantly (78, 79). Thus, the increased expression levels of PAXX in TMZ-resistant cells indicated that PAXX might contribute to TMZ resistance *via* BER pathway. In order to verify this conjecture, a GFP-based reporter assay was used to detect BER efficiency in PAXX-deficient cells, and results showed that the BER efficiency was reduced by 50% in PAXX-deficient U87 cells. Previous studies had identified that polymerase beta (pol β) was crucial for BER pathway, and knocking out pol β caused significant BER reduction (78, 80). Consistently, PAXX interplayed with pol β *in vitro*, indicating that PAXX might contribute to BER *via* interacting with pol β, which conduced TMZ drug resistance in glioma cells (75).

Interestingly, HAP1 cells that contained one single copy of almost every human chromosome knocked out by PAXX were less sensitive to DNA damage reagents such as zeocin and etoposide, with no significant change in genome stability (44). These results suggested that PAXX may have an extremely complex regulatory mechanism under physiological conditions in different cell lines.

**Figure 3:**
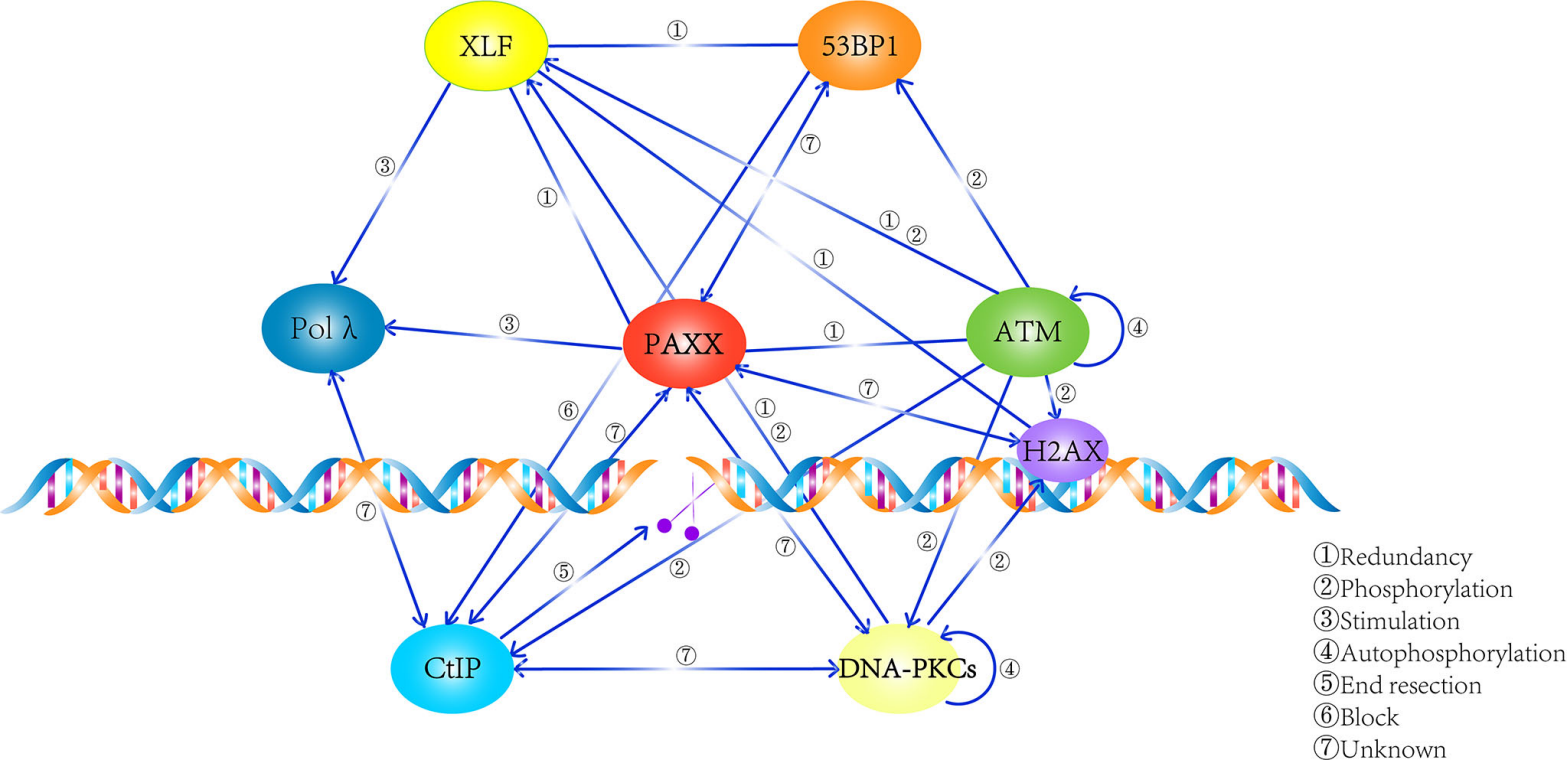
Summary of the network that PAXX participates in. PAXX plays an essential role in mediating the process of DNA repair, while the relationship among these factors were described.

In summary, these findings enlarge the dimensions of the PAXX functions: it not only plays an important role in cNHEJ but also contributes to BER and would not be surprising in other repair pathways such as MMEJ. Thus, the mechanism that PAXX plays roles in these cNHEJ-independent repair pathways requires to be further determined (**Figure 3**).

## Role of PAXX in Mammalian Development

cNHEJ is essential for maintaining the genomic stability and the development of the mammalian immune system. The deletion or mutation of the cNHEJ factors causes abnormal V(D)J recombination or CSR, leading to defects in lymphocyte development. Defects in cNHEJ factors were also observed in human patients. For example, patients with XLF deficiency usually have microcephaly and growth delay (81). Artemis null-deletion patients lacks T and B cells, and patients with abnormally low expression have spontaneous EBV-associated leukemia and progressive immunodeficiency (82, 83). Patients with DNA-PKcs deletion have microcephaly, developmental delay, and postnatal neurodegeneration (84).

To assess the physiological functions of PAXX in mammalian development, several groups including us generated PAXX-deficient mice. Unexpectedly, PAXX-deficient mice were viable and grew normally. Knocking out of *Paxx* gene does not affect the weight, size, fecundity, or development of the central nervous system (47, 85). Besides, the immune systems of the mice have been extensively analyzed. The spleen, thymus, bone marrow, and lymphocyte development showed no significant difference with that of WT mice, in terms of the organ appearance and flow cytometry analysis of the surface markers (36, 47, 85). In addition, no significant role of PAXX was observed in genome stability maintenance, V(D)J recombination, or CSR in PAXX-deficient mice (47, 85).

Since double deficiency of two cNHEJ factors or one cNHEJ factor with other DNA-repair-associated factor in mouse models exhibited both synthetic viability (KU and LIG4) or synthetic lethality (XLF and H2AX) (86); Balmus et al. and other labs crossed the *Paxx* ^−/−^ mice into ATM-, KU80-, LIG4-, or XLF-deficient backgrounds. *Paxx^−/−^Atm^−/−^* mice were born at frequencies similar to *Atm* ^−/−^ mice and did not show any overt phenotypes compared with *Ku80^−/−^* mice. Similarly, *Paxx^−/−^Ku80^−/−^* mice displayed no additional phenotypes compared with *Ku80^−/−^* mice. In addition, after multiple rounds of breeding, no viable *Paxx^−/−^Lig4^−/−^* offspring was observed, indicating that PAXX deficiency cannot rescue the embryonic lethality caused by LIG4 depletion. These results suggested that deficiency of PAXX was epistatic with ATM, KU, and LIG4 deficiency in mammalian development (36). However, it is not quite clear whether PAXX has redundant functions with other DNA repair factors such as 53BP1, given the fact that both genes located on the same chromosome in mice. Surprisingly, PAXX and XLF double-deficient mice are lethal during embryonic development (36, 47). *Xlf^−/−^Paxx^−/−^* embryos were observed at the expected Mendelian ratios in E14.5, but the size was smaller than the littermate controls. However, by E18.5, the *Xlf^−/−^Paxx^−/−^* embryos were no longer obtained at the expected rates. A few survival embryos showed reduced body weight and smaller spleen and thymus. Similar to XRCC4-deficient mice that showed severe neuronal apoptosis, the brain of the *Xlf^−/−^Paxx^−/−^* mice contained significant increased number of apoptotic inclusions in the post-mitotic intermediate zone, which might be the cause of the embryonic death. Consistently, in both E10.5 and E14.5, an increase in the number of γH2AX-positive cells was observed in the central nervous system (CNS) of the *Xlf^−/−^Paxx^−/−^* mice, compared with the WT littermates. These results revealed a critical role of PAXX in the survival neurons and embryonic development in XLF-deficient mice. Recently, Castañeda-Zegarra et al. showed that p53 deficiency rescued embryonic lethality of *Xlf^−/−^Paxx^−/−^* mice, the *Xlf^−/−^Paxx^−/−^ p53^−/−^* mice lived up to 60 days and died for unknown reasons without tumors. In addition, *Xlf^−/−^Paxx^−/−^ p53^+/−^* mice were also live-born and possessed reduced body weight, reduced size of spleens and thymus, and severe lymphocytopenia (87). Conditional knockouts may help us to further study the physiological function of PAXX in specific tissues. In fact, Musilli et al. conditionally knocked out XLF in hematopoietic stem cells or mature B cells in PAXX knockout mice and confirmed that PAXX and XLF are redundant in the V(D)J recombination, but not in CSR (88). This result was consistent with the results performed in the CH12F3 B-cell lymphoma cell line based CSR assay showing that PAXX was dispensable for CSR cNHEJ in the presence or absence of XLF (53).

In summary, present data suggested a very important role of PAXX in mouse neuron and lymphocytes development that was masked by XLF.

## Role of PAXX in V(D)J Recombination and B-Cell Development

cNHEJ is required for V(D)J recombination during the lymphocyte development and is important for class switch recombination (CSR) process (89, 90). Deletion of cNHEJ factors such as LIG4, XRCC4, DNA-PKcs, and Artemis leads to defects in V(D)J recombination, resulting in severe combined immunodeficiency (SCID) (91). In contrast, PAXX-deficient mice had normal V(D)J recombination and CSR, and the number and proportion of T- and B-lymphocytes are comparable to those of WT mice (47). 5′ RACE RT-PCR and next-generation sequencing revealed that PAXX mice had a well-diversified TCRα repertoire, comparable to WT mice (48).

However, *Xlf^−/−^Paxx^−/−^* embryos exhibited a 10-fold decrease in thymocyte number, mainly accounted for the CD4^+^CD8^+^ double-positive (DP) cells. Analysis of the existing CD4^−^CD8^−^ double-negative (DN) thymocytes showed that the CD44^−^CD25^+^CD28^+^ cells, which had undergone successful rearrangement of the TCR-β locus, were significantly reduced in *Xlf* ^−/−^ *Paxx* ^−/−^ E18.5 embryos. Consistently, PCR assay that detected TCR-β rearrangements involving Vβ10 and the Dβ2-Jβ2 cluster was performed, and no products were found in DNA from *Xlf* ^−/−^ *Paxx* ^−/−^ thymocytes. This observation indicated an impairment of V(D)J recombination at the TCR-β locus in thymocytes from *Xlf* ^−/−^ *Paxx* ^−/−^ embryos.

As for B-cell development, V(D)J recombination took place in the fetal liver (FL) at around E17.5 at which time μH chain became detectable after successful rearrangement (92). Whereas ~20% of pro-B cells expressed the intracytoplasmic μH chain (iIgM) in FL of littermates *Xlf^+/−^ Paxx^+/−^* embryos, only approximately 5% of this population was observed in the *Xlf* ^−/−^ *Paxx* ^−/−^ embryos, implying major defects in rearrangements (48). Conditional knockout of Xlf in Paxx^−/−^ mice showed similar results that recapitulate the profound immune deficiency caused by a block in the V(D)J process noted previously in E18.5 PAXX/XLF double knockout fetuses (88). Not surprisingly, CD21-Cre-mediated deletion of Xlf in *Paxx^−/−^* mature B cells showed a similar CSR defect compared with *Xlf^−/−^* B cells, excluding the role of PAXX in this particular stage during B-cell development.

## PAXX and Cancer Development

Aberrant DNA damage repair is a major cause of genomic instability, and the latter is the most common feature of human tumor formation. Therefore, it is reasonable to ask whether PAXX is associated with cancer genesis. Unfortunately, we and other groups did not observe early spontaneous tumor development in Paxx^−/−^ mice (47, 88). Consistently, lymphocytes and MEF cells from PAXX-deficient mice did not show severe genomic instability. However, although PAXX itself does not seem to play a role in tumor suppression, it is still possible that loss of PAXX might reduce the fidelity of the end-joining products of cNHEJ, which in turn may cause abnormal gene expression and promote cancer development in the late stage of the lifespan. Furthermore, PAXX-deficient cells were more sensitive to ionizing radiation. This might be because PAXX loss was prone to accumulate more harmful mutations, which may eventually lead to cell death, or evolved to the other extreme—immortality. Meanwhile, it is not clear whether PAXX deficiency would accelerate tumorigenesis in tumorigenesis models such as p53-deficient or KRAS mice.

PAXX single knockout has no significant effect on mouse development (85), but PAXX/XLF double knockout mice show severe genomic instability and neuronal cell apoptosis and eventually lead to embryonic death (48). Therefore, in PAXX knockout mice, XLF can rescue most of the biological defects caused by the loss of PAXX. Intriguingly, in colon cancer patients, Xlf expression was negatively correlated with PAXX expression. Hence, whether the role of PAXX in tumorigenesis is hidden in the presence of XLF is also to be explored, which could only be accessed by generating conditional knockout mouse models. In a mouse model generated in which Cre was expressed under control of the iVav promoter, Xlf was conditionally knocked out in a Paxx^−/−^ background, turned as XlfΔiVav mice. Mortality of the XlfΔiVav mice was sharply increased compared with the Paxx^−/−^ mice. In addition, XlfΔiVav mice showed decreased weight and moderate to severe alopecia. More importantly, three of five analyzed mice developed a thymic mass composed of cells that had larger than normal nucleus, showing the tumorigenic nature. Further flow cytometric analysis showed increased proportion of CD44+ CD62L+ cells with negative TCRβ+ staining. These observed results suggested that, at least in in hemopoietic system, PAXX prevents tumorigenesis in XLF-deficient background in aging mice.

It is also not clear whether the mutation of PAXX had some gain of function in tumorigenesis. This is not surprising since protein kinases ATM, DNA-PKcs, and ATR, which play essential roles in DNA repair, all have unexpected structural function (93, 94).

Surprisingly, different from what was predicted in mouse model, PAXX was found to be highly expressed in the TCGA colon cancer dataset due to the hypomethylation status of the PAXX gene promoter region in tumor cells. In addition, some studies have shown that PAXX was highly expressed in several drug-resistant cancer cells, such as osteosarcoma (OS) and glioma cells. Even though the underlying mechanism was not quite known, it was suggested that PAXX may be involved in some proto-carcinoma signaling pathways through its function beyond DNA repair. Meanwhile, there is a correlation between the overexpression of PAXX and the survival rate of patients, and the expression of PAXX may be used as a prognostic marker for disease-specific survival (95).

At present, multi-channel target combination therapy has received more and more attention and has achieved ideal clinical effects, such as poly(ADP-ribose) polymerase (PARP) inhibitors for the treatment of HR-deficient tumors (96, 97). Interestingly, in OS cells that are resistant to doxorubicin or cisplatin, the expression of PAXX was upregulated, and when PAXX was knocked out or specifically inhibited, drug sensitivity was restored. In this scenario, PAXX might be used as a target to effectively promote the effects of chemotherapy drugs (95). In addition, for tumors with XLF mutations, we could inhibit PAXX,which has less damage to the body after knockdown, as adjuvant therapy. It can not only improve the lethality of radiotherapy/chemotherapy to tumors but also effectively reduce the damage of radiotherapy/chemotherapy to normal cells, thereby achieving the effect of improving the sensitivity of tumor radiotherapy/chemotherapy and reducing toxic side effects. In addition, in view of the drug resistance of tumors with high expression of PAXX, whether the drug resistance can be reversed by finding suitable targets and designing combination drugs will play an important role in improving the efficiency of tumor treatment. Therefore, in combination with the biological functions of PAXX, the design of small molecule inhibitors targeting PAXX and the way of combination drugs are still scientific issues worth exploring.

## Discussion and Future Direction

### Post-Translational Modifications of PAXX

Posttranslational modification (PTM) is an important regulator of protein functions including its activity, stability, subcellular localization, and interaction with other proteins. These modifications include but not limited to phosphorylation, methylation, acetylation, glycosylation, ubiquitination, ufmylation, nitrosylation, and lipidation (98–100).

Phosphorylation of cNHEJ factors play critical roles in the regulation of the repair efficiency. For example, Liu et al. found that Akt kinase phosphorylated XLF at T181 to trigger its dissociation from the LIG4/XRCC4 complex. Phosphorylated XLF then interacted with 14-3-3β and localized to cytoplasm. The cytosolic XLF was eventually degraded by Skp, Cullin, and F-box containing complex (SCF complex) (101). Another example is that ATM trans-phosphorylated DNA-PKcs to regulate end-processing by promoting Artemis recruitment. Meanwhile, autophosphorylation of DNA-PKcs was necessary to relieve the physical blockage of the DNA-PKcs protein itself at the DNA ends (24).

Preliminary studies have shown that phosphorylation of PAXX tail (S134, T145, S148, and S152) might play a role in maintaining the stability of the PAXX-DNA-KU ternary complex, but the underlying mechanisms have yet to be studied (38). In addition, it is not clear which kinase(s) are responsible for catalyzing these sites and under which circumstances. It is notable that PAXX does not play a synergistic role with ATM in cNHEJ, but each has overlapping functions with XLF. In this regard, PAXX and ATM may each has distinct redundant functions with XLF, or alternatively, PAXX may operate downstream of ATM in end joining. In other words, it is possible that PAXX is a substrate of ATM, and its function is dependent on ATM activity. Surprisingly, in some cases, phosphorylation might not be necessarily a determining factor regulating DNA repair. Yu et al. identified two major phosphorylation sites (S245 and S251) of XLF in the C-terminus of the protein. *In vivo* experiments showed that S245 and S251 were phosphorylated by DNA-PK and ATM, respectively. However, phosphorylation of these two sites did not have a significant effect on the ability of XLF to interact with DNA or to be recruited to the DNA damaging site. S245A/S251A mutants could restore the DSB repair defect and radio-sensitivity in XLF-deficient 2BN cells (102). In the case of PAXX, besides the phosphorylation sites mentioned above, there could be other phosphorylation sites that were not discovered but play essential roles in regulating PAXX functions.

In addition, PAXX could be regulated by other types of post-translational modifications such as methylation, acetylation, and ubiquitination. We hypothesize that different types of modifications could have crosstalk with each other and essentially finetune PAXX function under different circumstances. Thus, exploring how is PAXX modified and how these modifications affect its function in DNA damage repair and other biological effects will help deepen the understanding of the biological mechanism of PAXX.

### Other Functions of PAXX Beyond DNA Repair

When the organism senses the DNA damage signal, it will initiate a series of signal transduction pathway networks to monitor, identify, and transmit the DNA damage signal, including changes in local chromatin conformation, stagnation of transcription activity, and activation of cell cycle checkpoints, thereby suspending the cell cycle processes and driving DNA repair (103–105). In this process, a variety of different biological processes are dynamically coordinated to regulate the process of DNA damage repair. As a regulator of NHEJ, the mechanism by which PAXX participates in the process of cNHEJ has not yet been determined.

It is also worthwhile to explore whether it functions in the above-mentioned biological processes beyond end joining. As we discussed previously, besides the association with other repair factors, such as KU70/80, LIG4/XRCC4, XLF, DNA-PKcs, Pol λ, and Pol β (**Table 1**), interactome analysis also revealed that PAXX connected with a variety of NHEJ accessory factors including PNKP, APTX, WRN, and PARP1, and other proteins such as multiple dynamin members (DYN1, 2, and 3) and TRF2/TERF2 and its interacting protein TERF2IP/RAP1 (37). Thus, how is PAXX’s participation in these pathways and molecular signals related to mammalian development remains an interesting question to be further explored.

**Table 1:** Paxx associated proteins.

Proteins	Genes	Functions	Phenotypes of the mice	References
Ku70/80	Xrcc6/Xrcc5	Recognizing and binding to the broken ends of the DNA and protecting them from further resection	1: Sick	https://www.sciencedirect.com/science/article/pii/S1383574214000386
Lig4/XRCC4	Lig4/Xrcc4	The LIG4-XRCC4 subcomplex is responsible for the NHEJ ligation step and XRCC4 enhances the joining activity of LIG4	1: lethal	https://www.sciencedirect.com/science/article/pii/S1097276500802646?via%3Dihub
XLF	Nhej1	Required for double-strand break (DSB) repair and V(D)J recombination	1: viable 2: Class Switch Recombination Defect	https://www.sciencedirect.com/science/article/pii/S1097276508005340
DNA-PKCs	Prkdc	Serine/threonine-protein kinase that acts as a molecular sensor for DNA damage	1: Viable 2: End processing problem	https://pubmed.ncbi.nlm.nih.gov/25818648/
Pol λ/Pol β	Poll/Polb	Repair polymerase involved in base excision repair (BER)	1: viable 2: Sensitivity of single and double knockout to DNA damaging agents	https://www.ncbi.nlm.nih.gov/sites/ppmc/articles/PMC2923601/

Numerous studies have shown that histone modifications and the corresponding enzymes have essential roles in DNA damage repair. In response to DSBs, histone methyltransferase G9a was phosphorylated and recruited to chromatin and then interacted with RPA to promote loading of the RPA and Rad51 to DSBs sites and eventually facilitate HR repair (106). Another example is that G9a-like protein (GLP) catalyzed H4K16me1 and facilitated the recruitment of 53BP1 to DNA DSBs (107). In the other way around, it is not quite clear whether the defects of the cNHEJ factors including PAXX have any roles in the change of the histone marks or the recruitment of the modification enzymes.

Ochi et al. showed that PAXX deficiency did not affect CHK1, CHK2, and H3S10 phosphorylation 1–8 h after 5 Gy irradiation in RPE-1 cells and concluded that PAXX loss did not impair checkpoint signaling. p53 activation after IR also seemed to be normal by checking the p21 expression levels in Paxx^−/−^ cells compared with WT cells (43). Interestingly, loss of DNA repair factor 53BP1 also exhibited a normal p53 activation in response to doxorubicin. However, when the cells were treated with centrinone, a potent specific Plk4 inhibitor to induce progressive loss of centrosomes as cells divide, p53 was activated in WT but not in the 53BP1^−/−^ cells. Thus, 53BP1 plays an important role in p53 activation and G1 arrest after centrosome loss or extended mitotic duration (108). Thus, it is reasonable to ask whether PAXX also has unique functions in special biological progress such as mitosis or cell division.

Even though PAXX is a cNHEJ factor, Trigg et al. observed that PAXX was both nuclear and cytoplasmic in U2OS cells. The nuclear PAXX was reduced after HSV-1 infection, but the cytoplasmic PAXX remained unchanged. This observation suggested a different role of nuclear and cytoplasmic PAXX in response to infection. In addition, they proved that PAXX restricts HSV-1 infection by reducing the production of infectious virions (109). Whether the anti-virus function of PAXX is related to its function in DNA repair is unknown. In fact, different from PAXX, LIG4 and XRCC4 promote HSV-1 replication (110), implying that PAXX plays a distinct role during HSV-1 infection. It is also not yet clear whether PAXX has an inhibitory effect on other viruses.

In summary, an in-depth understanding of the biological processes involved and clarification of its related regulatory mechanisms will help us to understand the functions of PAXX more comprehensively and will eventually shed light on the therapeutic strategies in the future.

## Author Contributions

JT and XL conceived and wrote the manuscript. JT, ZL, QW, MI, WL, and XL read and approved the final manuscript. All authors contributed to the article and approved the submitted version.

## Funding

This work was supported by the Guangdong Provincial Key Laboratory of Regional Immunity and Diseases (Grant Number 2019B030301009), National Natural Science Foundation of China (81972661), and high-level university phase 2 construction funding from Shenzhen University (860-00000210).

## Conflict of Interest

The authors declare that the research was conducted in the absence of any commercial or financial relationships that could be construed as a potential conflict of interest.

## Publisher’s Note

All claims expressed in this article are solely those of the authors and do not necessarily represent those of their affiliated organizations, or those of the publisher, the editors and the reviewers. Any product that may be evaluated in this article, or claim that may be made by its manufacturer, is not guaranteed or endorsed by the publisher.
